# Impact of Reclassification on Thyroid Nodules with Architectural Atypia: From Non-Invasive Encapsulated Follicular Variant Papillary Thyroid Carcinomas to Non-Invasive Follicular Thyroid Neoplasm with Papillary-Like Nuclear Features

**DOI:** 10.1371/journal.pone.0167756

**Published:** 2016-12-09

**Authors:** Min Ji Jeon, Dong Eun Song, Chan Kwon Jung, Won Gu Kim, Hyemi Kwon, Yu-Mi Lee, Tae-Yon Sung, Jong Ho Yoon, Ki-Wook Chung, Suck Joon Hong, Jung Hwan Baek, Jeong Hyun Lee, Tae Yong Kim, Young Kee Shong, Won Bae Kim

**Affiliations:** 1 Department of Internal Medicine, Asan Medical Center, University of Ulsan College of Medicine, Seoul, Korea; 2 Department of Pathology, Asan Medical Center, University of Ulsan College of Medicine, Seoul, Korea; 3 Department of Hospital Pathology, College of Medicine, The Catholic University of Korea, Seoul, Korea; 4 Department of Surgery, Asan Medical Center, University of Ulsan College of Medicine, Seoul, Korea; 5 Department of Radiology and Research Institute of Radiology, Asan Medical Center, University of Ulsan College of Medicine, Seoul, Korea; Catalan Institute of Oncology, SPAIN

## Abstract

**Background:**

The follicular variant of papillary thyroid cancer (FVPTC), especially the encapsulated non-invasive subtype, is a controversial entity. Recent study suggested using ‘non-invasive follicular thyroid neoplasm with papillary-like nuclear features (NIFTP)’ for these indolent carcinomas. We evaluated the impact of reclassification from non-invasive encapsulated FVPTCs (EFVPTCs) to NIFTPs in the diagnosis of thyroid nodules with architectural atypia.

**Methods:**

We reviewed 1301 thyroid nodules with architectural atypia in core needle biopsy (CNB) specimens obtained from March 2012 to February 2013. Nodules were classified into atypia of undetermined significance with architectural atypia (AUS-A, 984, 76%) or follicular neoplasm/suspicious for a follicular neoplasm (FN/SFN, 317, 24%). Among them, diagnostic surgery was performed in 384 nodules (30%).

**Results:**

In total, 160 nodules (42%) presented final malignant diagnoses including 39 non-invasive encapsulated FVPTCs (10%). The malignancy rate was estimated to be 7–35% in AUS-A nodules and 28–49% in FN/SFN nodules. After reclassification, the malignancy rate was much decreased and estimated to be 5–24% in AUS-A nodules, and 23–39% in FN/SFN nodules. Thyroid nodules with final malignant diagnoses were significantly more likely to have a FN/SFN CNB diagnosis, malignant US features and concomitant nuclear atypia in CNB specimens. However, these factors could not differentiate NIFTPs from other malignancies.

**Conclusions:**

After reclassification of non-invasive EFVPTCs to NIFTPs, the malignancy rate of thyroid nodules with architectural atypia in CNB specimens was decreased. However, there were no preoperative factors differentiating other malignancies from NIFTPs. The presence of malignant US features or concomitant nuclear atypia might help clinicians deciding diagnostic surgery but, these features also might indicate NIFTPs.

## Introduction

The follicular variant of papillary thyroid cancer (FVPTC) is a one common subtype of thyroid cancer. There are two main subtypes of FVPTCs: infiltrative and encapsulated. FVPTC, especially the encapsulated non-invasive subtype, is a controversial entity with low inter-observer reproducibility, and only small percentages of these tumors are reported to show aggressive clinical behavior [1]. Because of the clinically indolent nature of non-invasive encapsulated FVPTCs (EFVPTCs), there was a suggestion that these tumors should no longer be termed as carcinomas, but be diagnosed using an alternative term, a non-invasive follicular thyroid neoplasm with papillary-like nuclear features (NIFTP) [1–3].

FVPTCs show both architectural atypia of neoplastic microfollicular proliferation more than 50% and nuclear atypia of papillary thyroid cancer with variable degree in diagnostic specimens and most of them resulted in indeterminate diagnostic categories in the thyroid fine needle aspiration biopsies [3] or core needle biopsy (CNB) specimens.

In this study, we evaluated the final pathologic diagnoses of thyroid nodules with indeterminate results, especially with architectural atypia, in CNB specimens. We aimed to analyze the changes in the malignancy rates of thyroid nodules with architectural atypia after reclassification of non-invasive EFVPTCs to NIFTPs. We also tried to find preoperative factors predicting final diagnoses of these nodules.

## Methods

### Thyroid nodules and patients

From March 2012 to February 2013, 3376 thyroid nodules were evaluated by US-guided CNB at the Asan Medical Center (Seoul, Korea). We excluded nodules evaluated with repeated CNB during the study period. Among the assessed thyroid nodules, 1301 (39%) presented with architectural atypia in the CNB specimens. We retrospectively reviewed the clinicopathological characteristics of these 1301 thyroid nodules and the patients. This study was approved by our institutional review board (2015–0905). Informed consent was waived due to the retrospective nature of this study and anonymized medical records were used for analysis.

### Ultrasonography and CNB

All thyroid US examinations were performed using an HDI 5000 or IU22 scanner (Philips Medical Systems, Bothell, WA) with a 12.5 MHz linear phased-array transducer. US-guided CNB procedures were performed by experienced radiologists (J.H.B. and J.H.L) or by residents or fellows under their supervision using a 1.1 cm or 1.6 cm excursion, 18-gauge, double-action, spring-activated needle (TSK Ace-cut, Create Medic, Yokohama, Japan). The size of the nodule was measured and the vessels along the approach route were evaluated by power Doppler US. After induction of local anesthesia, the needle was advanced into the solid part of a nodule. After measuring the firing distance, the stylet and cutting cannula of the needle were sequentially fired. Tissue cores were immediately placed in 10% buffered formalin and processed. After the CNB, each patient was observed with a firm, local compression for 20 minutes [4–7].

The US findings for thyroid nodules were evaluated for the following features: size (maximal diameter); internal composition (solid, predominantly solid, predominantly cystic, or cystic); shape (oval to round, taller than wide or irregular); margins (smooth, spiculated or ill-defined); echogenicity (isoechoic, hypoechoic, markedly hypoechoic or hyperechoic); and the presence of calcification (microcalcification, incomplete macrocalcification, complete macrocalcification or rim calcification) [8]. The US criteria for malignant nodules were a taller than wide shape, spiculated margin, markedly hypoechogenicity, and the presence of micro- or macrocalcification [9].

### Pathologic evaluation of CNB and diagnostic surgical specimens

All CNB specimens were reviewed and diagnosed by an experienced pathologist (D.E.S) based on the criteria proposed by the Korean endocrine pathology thyroid CNB study group [10]. Atypia of undetermined significance with architectural atypia (AUS-A) category were defined as microfollicular, solid, trabecular or Hurthle cells proliferative lesions lacking a fibrous tumor capsule or the adjacent non-lesional parenchyma in the CNB specimen. Thyroid nodules with architectural atypia and an equivocal tumor capsule in background of extensive stromal fibrosis, multinodular goiter or Hashimoto’s thyroiditis were diagnosed as AUS-A category. Follicular neoplasm/suspicious for a follicular neoplasm (FN/SFN) category was defined as microfollicular, mixed microfollicular and normofollicular, solid, trabecular or Hurthle cells proliferative lesions with a definite fibrous tumor capsule in the CNB specimen. Under these criteria, 984 thyroid nodules (76%) had AUS-A, and 317 thyroid nodules (24%) had FN/SFN CNB diagnoses. Mild nuclear atypia was defined as more subtle papillary cancer-type nuclear atypia than that of the classic type of PTC including at least two nuclear parameters: including (a) nuclear enlargement, (b) mild nuclear overlapping with oval nuclear shape, (c) irregular nuclear membrane with occasional nuclear grooves, and (d) chromatin clearing. We defined concomitant mild nuclear atypia in a background of architectural atypia when these nuclear features are observed in neoplastic follicular cells more than 50% in CNB specimens [1].

A total of 384 diagnostic surgical specimens were reviewed and diagnosed, based on the WHO classification criteria [11]. Histological diagnostic criteria of non-invasive EFVPTC (or NIFTP) included follicular growth pattern, encapsulation (completely or partially) with clear demarcation from adjacent thyroid parenchyma, presence of papillary cancer-type nuclear atypia, and absence of capsular or vascular invasion in all tumor capsule similar to the reported criteria by Nikiforov et al. [1]. EFVPTC with any capsular or vascular invasion in all tumor capsule was classified as invasive EFVPTC. Criteria for capsular invasion included tumor buds penetrating outer contour of the capsule and/or separate satellite tumor nest with identical tumor cells outside the capsule. Criteria for vascular invasion included polypoid tumor nest covered by endothelium, attached to wall of blood vessels located within or outside the capsule, rarely associated with a thrombus, and excluded free floating tumor nests in vascular lumens. FVPTC with any focal infiltrative border in the adjacent thyroid parenchyma was classified as infiltrative FVPTC.

Another experienced pathologist (C.K.J.) independently reviewed the pathologic slides when the diagnoses of CNB specimens or final pathologies were hard to make.

### Statistics

R studio version 0.98.1091 and the R libraries survival, pROC and gdata were used to analyze data (R Foundation for Statistical Computing, Vienna, Austria, http://www.R-project.org/). Continuous variables are presented as the medians with interquartile ranges (IQR) and categorical variables as numbers with percentages. The Wilcoxon signed-rank test, Kurskal-Wallis test, Cochran-Armitage trend test and Chi-square tests were used to compare the characteristics of patients and nodules according to classification. The malignancy rates of thyroid nodules were estimated using the range. The low limit was calculated by dividing the number of malignancies confirmed in the final pathology by the total number of nodules evaluated by CNB. The upper limit was calculated by dividing the number of malignancies confirmed in the final pathology by the number of nodules that underwent diagnostic surgery [12]. All *P* values are 2-sided, with *P* < 0.05 considered statistically significant.

## Results

### Baseline characteristics of patients and thyroid nodules with architectural atypia

In total, 1301 thyroid nodules from 1253 patients were included in this study. The median age of the patients was 53 years (IQR 44–61), and 994 patients (79%) were female. CNB was used as a 1^st^ line diagnostic tool in 1056 thyroid nodules (81%) and as a 2^nd^ line tool in 245 thyroid nodules (19%) with previous non-diagnostic or indeterminate cytology results [11]. The median size of thyroid nodules was 1.9cm (IQR 1.2–2.8) and 101 thyroid nodules (8%) had concomitant mild nuclear atypia in a background of architectural atypia. In terms of US findings, 509 thyroid nodules (39%) had one or more malignant US features (Table 1).

**Table 1:** Baseline characteristics of the thyroid nodules with architectural atypia.

	Total	Nodules without final pathology	Nodules with final pathology	*P*-value
No. of nodules	1301	917 (70)	384 (30)	
CNB diagnoses				<0.001
AUS-A	984 (76)	784 (85)	200 (52)	
FN/SFN	317 (24)	133 (15)	184 (48)	
Size of nodules (cm)	1.9 (1.2–2.8)	1.6 (1.1–2.6)	2.4 (1.7–3.5)	<0.001
Presence of malignant US features				0.057
yes	509 (39)	343 (37)	166 (43)	
Presence of concomitant mild nuclear atypia				<0.001
yes	101 (8)	55 (6)	46 (12)	

CNB, core needle biopsy; AUS-A, atypia of undetermined significance with architectural atypia; FN/SFN, follicular neoplasm/suspicious for a follicular neoplasm; US, ultrasonography. Continuous variables are presented as medians with interquartile ranges. Categorical variables are presented as a number with percentages.

We considered diagnostic surgery in 577 patients, but 151 patients refused surgery and 59 patients had other serious medical problems. As a result, 367 patients underwent diagnostic surgery, and the final pathologic diagnoses were available in 384 thyroid nodules with architectural atypia (30%). Thyroid nodules that underwent diagnostic surgery were larger, more likely to have a FN/SFN CNB diagnosis, and have a concomitant nuclear atypia than nodules that did not undergo diagnostic surgery (Table 1).

### Final pathologic diagnoses of thyroid nodules with architectural atypia

The final pathologic diagnoses of 384 thyroid nodules with architectural atypia that were examined by diagnostic surgery are presented in Table 2. In total, 96 nodules (25%) were revealed to be non-neoplastic hyperplasias, 129 nodules (34%) follicular adenomas, and 159 nodules (42%) various malignant lesions, including 86 FVPTCs (22%), 64 minimally invasive follicular thyroid carcinomas (miFTCs, 17%), and 5 solid variants of papillary thyroid carcinoma (PTC), 2 clear cell variants of PTC, and 2 classical PTCs.

**Table 2:** Final pathologic diagnoses of the thyroid nodules with architectural atypia.

	Total	CNB results		*P*-value
		AUS-A	FN /SFN	
Non-neoplastic hyperplasia	96 (25)	83 (42)	13 (7)	< 0.001*
Adenoma	129 (34)	48 (24)	81 (44)	
Malignancy	159 (42)	69 (35)	90 (49)	
FVPTC	86 (22)	43 (22)	43 (23)	
Encapsulated				
Non-invasive	39 (10)	21 (11)	18 (10)	
Invasive	39 (10)	18 (9)	21 (11)	
Infiltrative	8 (2)	4 (2)	4 (2)	
miFTC	64 (17)	24 (12)	40 (22)	
other variants of PTC	9 (2)	2 (1)	7 (4)	

CNB, core needle biopsy; AUS-A, atypia of undetermined significant with architectural atypia; FN/SFN, follicular neoplasm/suspicious for a follicular neoplasm; PTC, papillary thyroid cancer; FVPTC, follicular variant PTC; miFTC, minimally invasive follicular thyroid cancer. Variables were presented as number with percentages.

*P value was estimated by Cochran-Armitage trend test.

**Fig 1:**
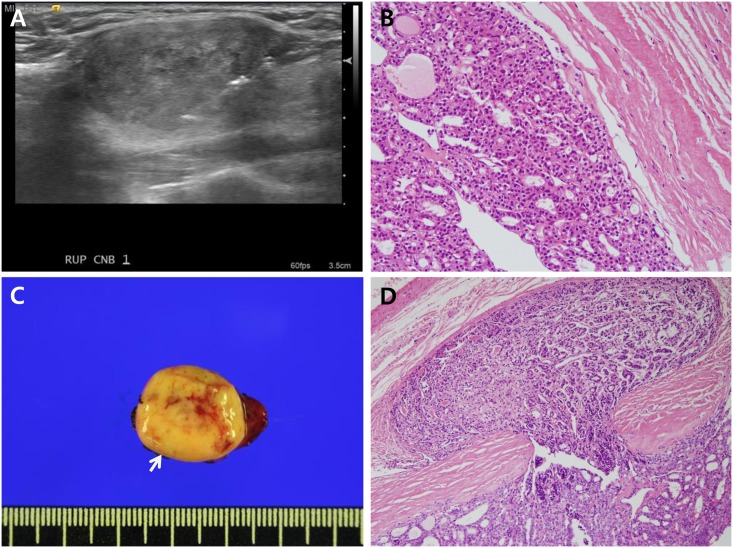
Radiologic and pathologic findings of the representative case. (**A**) Ultrasonography showed a 2.8 cm sized isoechoic thyroid nodule in the right upper lobe. This nodule was biopsied using spring-activated core needle. (**B**) The core needle biopsy specimen showed histological features of suspicious follicular neoplasm with microfollicular proliferation and thin tumor capsule. (**C-D**) Hemithyroidectomy specimen grossly revealed totally encapsulated 3 cm sized round nodule in the right lobe (C, arrow) and histologically demonstrated invasive encapsulated follicular variant papillary thyroid carcinoma with definite capsular invasion (**D**).

Of these 86 FVPTCs, 78 were the encapsulated subtype. Of 43 FVPTCs with AUS-A CNB diagnoses, 21 nodules (49%) were non-invasive EFVPTCs, 18 nodules (42%) were invasive EFVPTCs, 4 nodules (9%) were infiltrative FVPTCs. Of 43 FVPTCs with FN/SFN CNB diagnoses, 18 nodules (42%) were non-invasive EFVPTCs, 21 nodules (49%) were invasive EFVPTCs, and 4 nodules (9%) were infiltrative FVPTCs (Table 2). Radiologic and pathologic findings from the representative case were presented in Fig 1.

The final pathologic diagnoses were significantly different according to the CNB diagnosis. Nodules with an AUS-A CNB diagnosis were more likely to be a non-neoplastic hyperplasia. However, nodules with a FN/SFN CNB diagnosis were more likely to be a follicular adenoma or various malignancies (*P* for trend < 0.001).

### Malignancy rates of thyroid nodules with architectural atypia before and after reclassification

**Fig 2:**
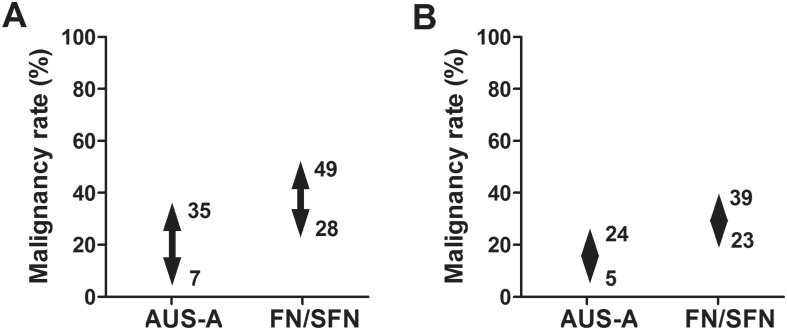
Malignancy rate of thyroid nodules with architectural atypia. (**A**) The malignancy rate was estimated to be 7–35% in AUS-A nodules and 28–49% in FN/SFN nodules. (**B**) After reclassification of follicular variant papillary thyroid carcinomas to non-invasive thyroid neoplasm with papillary-like nuclear features, the malignancy rate was estimated to be 5–24% in AUS-A nodules and 23–39% in FN/SFN nodules. (AUS-A, atypia of undetermined significance with architectural atypia; FN/SFN, follicular neoplasm/suspicious for a follicular neoplasm).

The malignancy rate was estimated to be 7–35% in AUS-A nodules and 28–49% in FN/SFN nodules (Fig 2A). Because 39 of 86 FVPTCs (45%) were non-invasive EFVPTCs, the malignancy rate much decreased when we reclassified non-invasive EFVPTCs to NIFTPs. In total, 121 nodules (31%) were malignant lesions including 47 FVPTCs (12%) after reclassification. The malignancy rate was estimated to be 5–24% in AUS-A nodules, and 23–39% in FN/SFN nodules after reclassification (Fig 2B).

### Predictable preoperative factors associated with final malignant diagnosis

We evaluated preoperative factors predicting malignancy in final diagnoses (Table 3). Thyroid nodules with final malignant diagnoses were significantly more likely to have a FN/SFN CNB diagnosis, malignant US features and nuclear atypia. The presence of malignant US features or nuclear atypia was only associated with PTCs, not with miFTCs (*P* < 0.001 or *P* < 0.001, respectively for PTCs; *P* = 0.326, *P* = 0.268, respectively for miFTCs, data not shown). After reclassification, thyroid nodules with final malignant diagnoses were also significantly more likely to have a FN/SFN CNB diagnosis, malignant US features and nuclear atypia. However, NIFTPs had similar preoperative radiologic and pathologic factors compared to thyroid nodules with final malignant diagnoses.

**Table 3:** Factors associated with malignancy of thyroid nodule with architectural before and after reclassification.

	Present classification		*P*-value	After reclassification			*P*-value
	Benign	Malignant		Benign	NIFTPs	Malignant	
No. of nodules	225 (59)	159 (41)		225 (59)	39 (10)	120 (31)	
Size (cm)	2.4 (1.7–3.4)	2.5 (1.7–3.6)	0.527*	2.4 (1.7–3.4)	2.3 (1.4–3.1)	2.7 (1.8–3.6)	0.089^‡^
CNB diagnoses			0.006^†^				0.003^§^
AUS-A	131 (58)	69 (43)		131 (58)	19 (49)	50 (42)	
FN/SFN	94 (42)	90 (57)		94 (42)	20 (51)	70 (58)	
Presence of malignant US features			<0.001^†^				<0.001^§^
Yes	81 (36)	85 (53)		81 (36)	20 (51)	65 (54)	
Nuclear atypia			<0.001^†^				<0.001^§^
yes	8 (4)	36 (23)		8 (4)	15 (38)	21 (18)	

NIFTP, non-invasive follicular thyroid neoplasm with papillary-like nuclear features. CNB, core needle biopsy; AUS-A, atypia of undetermined significant with architectural atypia; FN/SFN, follicular neoplasm/suspicious for a follicular neoplasm;US, ultrasonography. Variables were presented as number with percentages.

*P value was estimated by Wilcoxon signed-rank test.

^†^P value was estimated by Chi-square test.

^‡^P value was estimated by Kruskal-Wallis test.

^§^P value was estimated by Cochran-Armitage trend test.

We did subgroup analysis of FVPTCs and evaluated factors differentiating subtypes of FVPTCs preoperatively. However, none of radiologic and pathologic factors were associated with subtypes of FVPTCs. About half of non-invasive EFVPTCs or NIFTPs presented malignant US features and had concomitant nuclear atypia (Table 4).

**Table 4:** Association between preoperative factors and subtypes of follicular variant papillary thyroid carcinomas.

Subtypes of FVPTCs	CNB diagnoses		*P*-value*	Presence of nuclear atypia		*P*-value*	Presence of malignant US features		*P*-value*
	AUS-A	SFN/FN		No	Yes		No	Yes	
Encapsulated			0.616			0.368			0.148
non-invasive	21 (49)	18 (42)		24 (42)	15 (52)		19 (50)	20 (42)	
invasive	18 (42)	21 (49)		27 (47)	12 (41)		18 (47)	21 (44)	
Infiltrative	4 (9)	4 (9)		6 (11)	2 (7)		1 (3)	7 (15)	

CNB, core needle biopsy; AUS-A, atypia of undetermined significant with architectural atypia; FN/SFN, follicular neoplasm/suspicious for a follicular neoplasm; FVPTC, follicular variant papillary thyroid cancer; US, ultrasonography. Variables were presented as number with percentages.

*P value was estimated by Cochran-Armitage trend test.

## Discussion

In our current comprehensive analysis of the final pathologic diagnoses of thyroid nodules with architectural atypia in CNB specimens, we found a high malignancy which ranged 7–35% in AUS-A nodules and 28–49% in FN/SFN nodules. Overall, 42% of thyroid nodules among 384 nodules with final pathologic diagnoses resulted in a malignancy, and about 25% of such nodules were non-invasive EFVPTCs. The malignancy rate was much decreased when we reclassified non-invasive EFVPTCs to NIFTPs, as previous studies [13, 14]. The malignancy rate after reclassification was ranged 5–24% in AUS-A nodules and 23–39% in FN-SFN nodules. This rate was similar what previously the Bethesda System for Reporting Thyroid Cytopathology suggested [15]. Our data are the first to present changes in the malignancy rate of thyroid nodule with indeterminate preoperative diagnosis by CNB with the introduction of NIFTPs.

In our present series, clinicians were more likely to do diagnostic surgery in thyroid nodules with a FN/SFN CNB diagnosis than those with an AUS-A diagnosis and also in thyroid nodules with malignant US features or concomitant nuclear atypia. Actually, the thyroid nodules with a FN/SFN CNB diagnosis, malignant US features, or nuclear atypia were more likely to be malignant lesions [16, 17]. However, these preoperative histologic or radiologic findings were not associated with the subtypes of FVPTCs and about half of non-invasive EFVPTCs also presented malignant US features or concomitant nuclear atypia in CNB specimens [18].

Our present findings suggested that recently suggested new classification of non-invasive EFVPTCs to NIFTPs could decrease the malignancy rates of thyroid nodules with indeterminate preoperative results, especially with architectural atypia, but could not change the clinical decision for diagnostic surgery. The final diagnoses of thyroid nodules with architectural atypia in CNB specimens were heterogeneous with not negligible malignancy rates even after reclassification of non-invasive EFVTCs to NIFTPs, and only diagnostic surgery could differentiate subtypes of malignancy in these nodules. Reclassification of non-invasive EFVPTCs to NIFTPs would prevent postoperative overtreatment of thyroid nodules, and patients with large non-invasive EFVPTCs would not undergo completion thyroidectomy with/without radioactive iodine treatment anymore with the adoption of new terminology of NIFTPs in the future. Actually, two patients having thyroid nodules classified as non-invasive EFVPTCs or NIFTPs underwent completion thyroidectomy with radioactive iodine treatment in this study. Our data also indicated that clinicians should keep in mind that thyroid nodules showing architectural atypia in CNB specimens with concomitant nuclear atypia and/or malignant US features have higher malignancy rates than those without concomitant nuclear atypia and/or malignant US features but, are also associated with NIFTPs [3] after histological confirmation.

Our study had several limitations of note. First, this was a retrospectively designed study, and the final pathologic diagnoses of thyroid nodules were only available in 30% of our cases. We presented the malignancy rate of thyroid nodules as ranges but, these might have been skewed by a selection bias. A referral bias for patients in a tertiary center should also be considered, and it could have led to a high malignancy rate for thyroid nodules in this study. Second, we only evaluated thyroid nodules with architectural atypia in CNB specimens and our data could not be generally applied to all preoperative indeterminate diagnostic categories.

In conclusion, thyroid nodules with architectural atypia in a CNB specimen have a high malignancy rate with a high prevalence of EFVPTCs. After reclassification of non-invasive EFVPTCs to NIFTPs, the malignancy rate was much decreased. However, there were no predictable factors differentiating other malignancies from NIFTPs. Diagnostic surgery is still inevitable for accurate diagnosis of thyroid nodules with architectural atypia. The presence of malignant US features or concomitant nuclear atypia might help clinicians deciding diagnostic surgery but, these features also might indicate NIFTPs.

## Supporting Information

S1 File Data set of this study. (XLSX)
